# Eicosapentaenoic acid therapy is associated with decreased coronary plaque instability assessed using optical frequency domain imaging

**DOI:** 10.1002/clc.23185

**Published:** 2019-04-23

**Authors:** Takao Konishi, Daisuke Sunaga, Naohiro Funayama, Tadashi Yamamoto, Hironori Murakami, Daisuke Hotta, Masanori Nojima, Shinya Tanaka

**Affiliations:** ^1^ Department of Cardiology Hokkaido Cardiovascular Hospital Sapporo Japan; ^2^ Department of Cancer Pathology Hokkaido University Faculty of Medicine Sapporo Japan; ^3^ Center for Translational Research, The Institute of Medical Science Hospital The University of Tokyo Tokyo Japan

**Keywords:** eicosapentaenoic acid, optical frequency domain imaging, percutaneous coronary intervention, plaque instability

## Abstract

**Background:**

The relationship between eicosapentaenoic acid (EPA) therapy and coronary plaque stability assessed by optical frequency domain imaging (OFDI) has not been thoroughly described.

**Hypothesis:**

EPA therapy is associated with decreased plaque instability in patients undergoing percutaneous coronary intervention (PCI) using OFDI.

**Methods:**

Data on coronary artery plaques from 121 patients who consecutively underwent PCI between October 2015 and July 2018 were retrospectively analyzed. Of these patients, 109 were untreated (no‐EPA group), whereas 12 were treated with EPA (EPA group). Each plaque's morphological characteristics were analyzed using OFDI.

**Results:**

We used 1:4 propensity score matching for patients who received or did not receive EPA therapy before PCI. Baseline characteristics were balanced between both groups (age, sex, body mass index, diabetes mellitus, hypertension, dyslipidemia, chronic kidney disease, smoking, previous PCI or coronary artery bypass grafting, previous myocardial infarction, prior statin use, acute coronary syndrome, hemoglobin A1c level, low‐density lipoprotein cholesterol concentration, triglyceride concentration, and high‐density lipoprotein cholesterol concentration). OFDI data from 60 patients were analyzed in this study. The EPA group had significantly lower mean lipid index (818 ± 806 vs 1574 ± 891) and macrophage grade (13.5 ± 5.9 vs 19.3 ± 7.4) but higher mean minimum fibrous cap thickness (109.2 ± 55.7 vs 81.6 ± 36.4 μm) than the no‐EPA group (*P* = 0.010, 0.019, and 0.040, respectively). Multiple logistic regression analyses showed that prior EPA use was independently associated with lower lipid index and macrophage grade (*P* = 0.043 and 0.024, respectively).

**Conclusion:**

This OFDI analysis suggests that EPA therapy is associated with decreased plaque instability in patients undergoing PCI.

## 1 INTRODUCTION

Eicosapentaenoic acid (EPA) is a member of a group of n−3 polyunsaturated fatty acids derived from fish oil. Epidemiologic data suggest that long‐term intake of n−3 polyunsaturated fatty acids plays an important role in reducing adverse cardiovascular events.^1^ Moreover, some clinical studies suggested that administration of purified EPA for secondary and primary prevention lowers the total and ischemic cardiovascular mortality.^2^, ^3^ Optical coherence tomography (OCT) and optical frequency domain imaging (OFDI) are helpful intravascular imaging modalities that use the reflection of near‐infrared light to create images. Recently, many OCT and OFDI studies have reported the characteristics of unstable plaques, including plaque rupture,^4^, ^5^, ^6^ lipid‐rich plaques,^7^ thin‐cap fibroatheroma (TCFA),^8^, ^9^ cholesterol crystals,^10^, ^11^ macrophage accumulation,^12^, ^13^ and microchannels.^8^, ^14^ Several coronary computed tomography studies have shown that coronary plaques had lower plaque burden in patients receiving EPA therapy than in those not receiving EPA therapy.^15^, ^16^ Furthermore, intravascular ultrasound studies have indicated smaller coronary atherosclerotic plaque or lipid volume in patients on EPA therapy than in those not on EPA therapy.^17^, ^18^ However, no study has comprehensively examined whether EPA therapy is associated with decreased plaque instability in patients undergoing percutaneous coronary intervention (PCI) using OFDI. This study aimed to investigate the association between EPA therapy and coronary plaque instability in patients undergoing PCI using OFDI.

## 2 METHODS

### 2.1 Sample population and follow‐up

Data collected from 98 men and 23 women aged >30 years who consecutively underwent OFDI‐guided PCI between October 2015 and July 2018 at Hokkaido Cardiovascular Hospital in Japan were retrospectively analyzed. A total of 109 untreated patients were allocated to the no‐EPA group, whereas 12 patients treated with EPA for ≥1 month were assigned to the EPA group. Patients presenting with left main coronary artery disease (CAD) and cardiogenic shock were excluded.

This study was approved by the ethics committee of Hokkaido Cardiovascular Hospital and was conducted in accordance with the ethical principles of the Declaration of Helsinki for medical research involving human subjects. The committee approved the image analysis, using only the data which were gotten when performing PCI.

### 2.2 Coronary angiography

Coronary angiograms were analyzed by offline quantitative coronary angiography (GE ver. 5.10.1; Pie Medical Imaging BV, Maastricht, the Netherlands). Reference diameter, minimum lumen diameter, diameter stenosis, and lesion length were measured.

### 2.3 OFDI and analysis

An OFDI catheter (FastView; Terumo Corporation, Tokyo, Japan) was advanced using a 0.014‐in. guide wire with the help of a 6‐ or 7‐Fr guiding catheter, and the imaging core was placed at the distal site of the lesion. Images obtained using OFDI were obtained using a continuous flush of contrast media at a rate of 4 mL/s, and the OFDI wire was pulled back at a rate of 20 to 40 mm/s. Although OFDI was generally performed without dilation using a balloon catheter, the lesion was dilated using a small‐sized balloon if the OFDI catheter could not pass through the lesion because of severe stenosis. Aspiration thrombectomy was performed before OFDI for patients with acute coronary syndrome who did not show spontaneous recanalization. The plaque morphology of culprit lesions was studied. Following identification of the most stenotic lesion, 5‐mm proximal and distal lesions (total length: 10 mm) were examined. Cross‐sectional images were analyzed at every 1 mm for each OFDI parameter. OFDI analysis was performed by two independent investigators (Takao Konishi and R. K.) who were blinded to each patient's clinical course. In case of discordance between the investigators, a consensus reading was performed.

### 2.4 OFDI definitions

**Figure 1:**
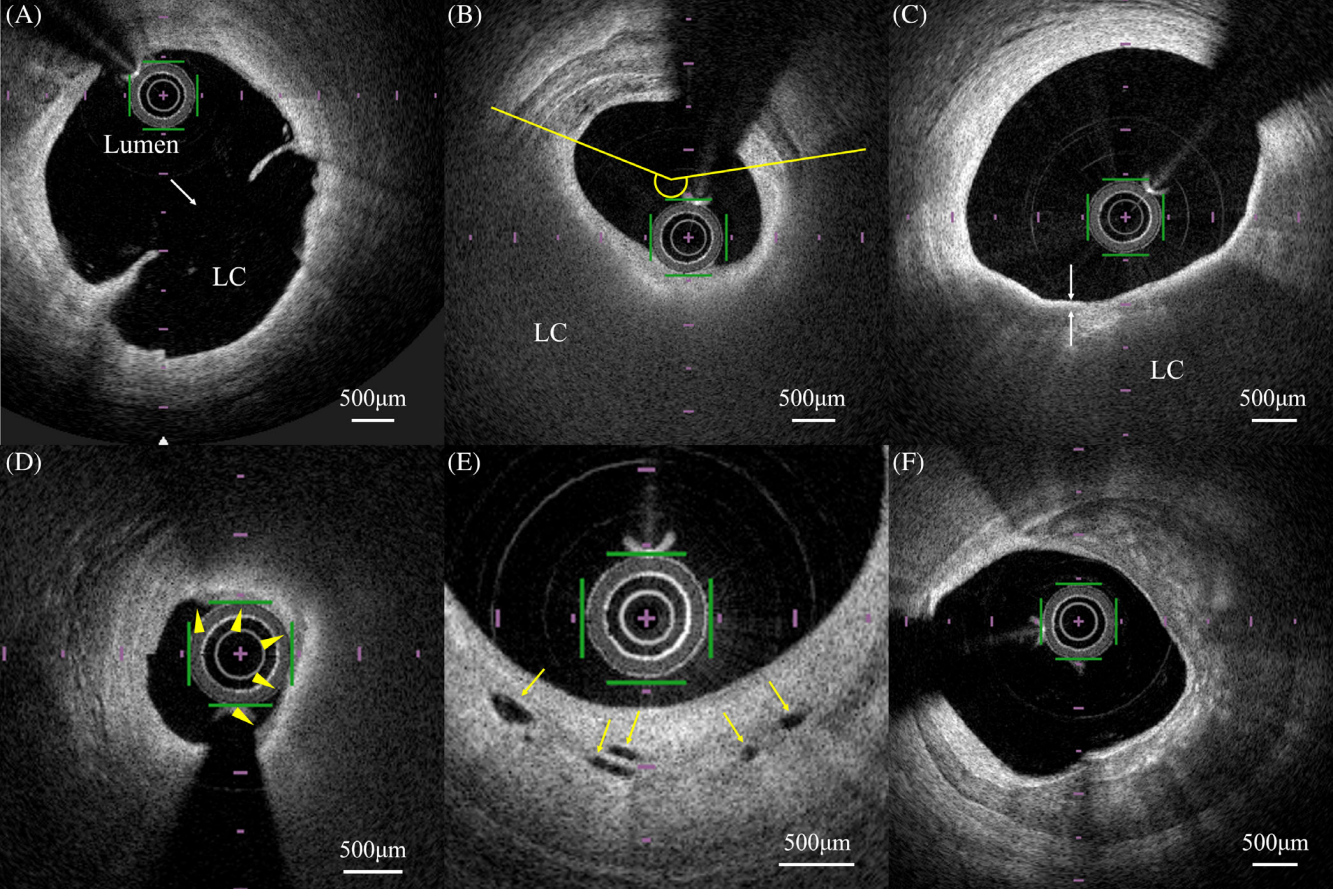
Representative coronary artery plaque images for optical frequency domain imaging (OFDI) analyses. A, Plaque rupture (white arrow) with fibrous cap discontinuity, leading to a communication between the LC and the lumen. LC, lipid core. B, Measurement of lipid arc in a lipid‐rich plaque. Maximum lipid arc was measured within a lipid‐rich plaque (yellow lines). C, Measurement of fibrous cap thickness (FCT). Minimum FCT was measured thrice at the thinnest part, and the three measurements were subsequently averaged (white arrows). D, Macrophage accumulation was defined as increased signal intensity within the fibrous cap, accompanied by heterogeneous backward shadows (arrowheads). E, Microchannels (yellow arrows) were defined as small vesicular or tubular structures with diameters of 50 to 300 μm within the intima. F, Calcification was defined as well‐delineated, low‐backscattering heterogeneous regions

OFDI analysis indicated the presence of plaque rupture, plaque erosion, calcified nodule, lipid‐rich plaque, TCFA, macrophage accumulation, microvessels, cholesterol crystals, and calcification in the plaque. Plaque rupture was defined as the presence of fibrous cap discontinuity leading to a communication between the necrotic core and lumen (Figure 1A).^19^, ^20^ Plaque erosion was defined as a lesion with a thrombus but without fibrous cap disruption.^21^ Thrombus was defined as a well‐delineated, high‐signal mass attached to the luminal surface or floating within the lumen.^20^ Lipid‐rich plaques were defined as lesions with a lipid arc of more than 180° (Figure 1B).^20^ Lipid arc was measured within a lipid‐rich plaque, and the maximum value was recorded (Figure 1B). Lipid‐core length was defined as the length of lipid plaque and was measured in the longitudinal view. Lipid index was defined as the maximum lipid arc multiplied by the lipid‐core length.^22^ Cholesterol crystals were defined as thin, linear regions of high signal intensity within the lipid plaque, without backscattering.^20^ TCFA was defined as an atheroma with a fibrous cap thickness (FCT) < 65 μm.^20^ FCT was defined as the minimum thickness of a signal‐rich layer from the coronary artery lumen to the inner border of the underlying lipid in the culprit lesion (Figure 1C).^20^ Macrophage accumulation was defined as increased signal intensity within the fibrous cap, accompanied by heterogeneous backward shadows (Figure 1D).^20^ Semiquantification of macrophage accumulation was performed as follows based on axial and circumferential distribution: grade 0, no macrophage; grade 1, localized macrophage accumulation; grade 2, clustered accumulation in <1 quadrant; grade 3, clustered accumulation in ≥1 quadrant and < 3 quadrants; and grade 4, clustered accumulation in ≥3 quadrants (Supporting Information Figure S1).^23^ The possible range of macrophage score was 0‐40, which is a summation of grades 0‐4 across all 10 slices. Microchannels were defined as small vesicular or tubular structures with diameters of 50‐300 μm within the intima (Figure 1E).^20^ The number of microchannels was counted across all 10 slices at every 1 mm. Calcification was defined as well‐delineated, low‐backscattering heterogeneous regions (Figure 1F).^20^

### 2.5 Statistical analysis

Propensity score‐matched analysis was performed to compare the two groups.^24^ The propensity of being in the EPA group was estimated using a logistic regression model with the following covariates in our study database: age, sex, body mass index, diabetes mellitus, hypertension, dyslipidemia, chronic kidney disease (CKD), smoking, previous PCI or coronary artery bypass grafting, previous myocardial infarction, prior statin use, acute coronary syndrome, hemoglobin A1c level, low‐density lipoprotein (LDL) cholesterol concentration, triglyceride concentration, and high‐density lipoprotein (HDL) cholesterol concentration. Each patient in the EPA group was matched to four patients in the no‐EPA group (1:4 matching), as there were more patients who did not receive EPA therapy. Greedy nearest‐neighbor matching without replacement was used.

Continuous and categorical variables are reported as means ± standard deviations and as counts and percentages, respectively. The normality of distributions was assessed using the Kolmogorov‐Smirnov test. Between‐group differences were analyzed using Pearson's *χ* ^2^ test or Fisher's exact test for categorical variables and Student's *t*‐test or Mann‐Whitney *U* test for continuous variables, as appropriate. A *P* value < 0.05 was considered to indicate statistical significance. Single and multiple logistic regression analyses were carried out to identify independent risk factors for coronary artery plaque characteristics assessed using OFDI. Risk factors that emerged with *P* values (Wald statistics) <0.05 in the single variable analysis were entered in the multiple variable regression analysis. Data were analyzed using spss statistical software version 25.0 (IBM Corporation, Armonk, NY, USA).

## 3 RESULTS

### 3.1 Clinical characteristics

After the propensity score matching, OFDI images from 60 patients were analyzed in this study. The clinical characteristics of the two patient groups are compared in Table 1. Although EPA/arachidonic acid ratios were available only in 31 patients (10 in EPA group and 21 in no‐EPA group, the mean EPA/arachidonic acid ratio was significantly higher in the EPA group than in the no‐EPA group (1.63 ± 0.46 vs 0.48 ± 0.21, *P* < 0.001). The mean LDL cholesterol concentration was 94 ± 29 mg/dL in the no‐EPA group and 97 ± 36 mg/dL in the EPA group (*P* = 0.807). Furthermore, the mean triglyceride concentration was 161 ± 92 mg/dL in the no‐EPA group and 153 ± 114 mg/dL in the EPA group (*P* = 0.803). Other characteristics including medications and concomitant diseases were similar in both groups. The type and doses of EPAs administered in 12 patients in the EPA group were as follows: ethyl icosapentate, 1800 mg/day (n = 5); ethyl icosapentate, 2700 mg/day (n = 1); and omega‐3‐acid ethyl esters, 2 g/day (n = 6).

**Table 1:** Baseline patient characteristics

	No‐EPA (n = 48)	EPA (n = 12)	*P* value
Age (years)	70.1 ± 9.2	71.3 ± 7.4	0.660
Male	37 (77)	10 (83)	0.638
Body mass index	24.5 ± 3.9	23.9 ± 1.9	0.451
Diabetes mellitus	18 (38)	7 (58)	0.190
Hypertension	41 (85)	9 (75)	0.403
Dyslipidemia	44 (92)	11 (92)	1.000
Chronic kidney disease (no hemodialysis)	10 (21)	2 (17)	0.747
Hemodialysis	6 (13)	1 (8)	0.688
Sleep apnea syndrome	5 (10)	1 (8)	0.830
Current smoker	3 (6)	1 (8)	0.796
Family history of coronary artery disease	3 (6)	1 (8)	0.796
History of PCI or CABG	28 (58)	8 (67)	0.746
History of myocardial infarction	15 (31)	6 (50)	0.312
History of TIA or cerebral infarction	7 (15)	2 (17)	0.857
History of peripheral artery disease	3 (6)	1 (8)	0.796
Prior statin use	35 (73)	9 (75)	0.884
Prior aspirin use	31 (65)	10 (83)	0.306
Prior clopidogrel use	22 (46)	9 (75)	0.071
Prior ACEI or ARB use	21 (44)	5 (42)	0.896
Prior calcium channel blocker use	25 (52)	5 (42)	0.519
Prior beta blocker use	14 (29)	5 (42)	0.493
Prior ezetimibe use	5 (10)	2 (17)	0.619
Duration of prior EPA use			
Duration <1 month		0	—
1‐12 months		9 (75)	—
≥12 months		3 (25)	—
Hemoglobin (g/dL)	13.2 ± 1.7	13.7 ± 1.5	0.416
HbA1c (%)	6.2 ± 0.8	6.5 ± 0.8	0.255
Glucose (mg/dL)	124 ± 36	125 ± 28	0.944
LDL‐C (mg/dL)	94 ± 29	97 ± 36	0.807
Triglyceride (mg/dL)	161 ± 92	153 ± 114	0.803
HDL‐C (mg/dL)	47 ± 10	50 ± 7	0.345
LDL‐C to HDL‐C ratio	2.2 ± 1.7	2.0 ± 0.9	0.628
EPA/AA ratio^a^	0.48 ± 0.21	1.63 ± 0.46	<0.001
Ca (mg/dL)	9.2 ± 0.3	9.4 ± 0.5	0.165
Acute coronary syndrome	9 (19)	1 (8)	0.670
ST‐segment elevation myocardial infarction	3 (6)	0	0.374
Non‐ST‐segment elevation myocardial infarction	6 (13)	1 (8)	0.688

*Note*: Values are presented as mean ± SD or number (%) of observations.

Abbreviations: AA, arachidonic acid; ACEI, angiotensin‐converting enzyme inhibitor; ARB, angiotensin II receptor blocker; CABG, coronary artery bypass grafting; EPA, eicosapentaenoic acid; HDL‐C, high‐density lipoprotein cholesterol; LDL‐C, low‐density lipoprotein cholesterol; PCI, percutaneous coronary intervention; TIA, transient ischemic attack.

a n = 31 (10 in EPA group and 21 in no‐EPA group).

### 3.2 Angiographic findings

Plaque location and angiographic data are shown in Table 2. Diameter stenosis was similar between the no‐EPA and EPA groups (85.9 ± 11.5 vs 86.4 ± 16.5, *P* = 0.885). No significant differences in plaque location and other angiographic data were observed between both groups.

**Table 2:** Angiographic and procedural characteristics

	No‐EPA (n = 48)	EPA (n = 12)	*P* value
Plaque location			
LAD, n (%)	31 (65)	4 (33)	0.102
LCX, n (%)	2 (4)	3 (25)	0.050
RCA, n (%)	15 (31)	5 (42)	0.511
Minimum lesion diameter, mm	1.01 ± 0.56	0.96 ± 0.54	0.763
Reference diameter, mm	3.02 ± 0.52	3.12 ± 0.50	0.578
Lesion length, mm	19.7 ± 9.6	18.5 ± 9.0	0.691
Diameter stenosis, %	85.9 ± 11.5	86.4 ± 16.5	0.885
Pre‐dilatation, n (%)	11 (23)	3 (25)	0.879
Aspiration thrombectomy, n (%)	3 (6)	0	0.374

Abbreviations: LAD, left anterior descending artery; LCX, left circumflex artery; RCA, right coronary artery.

### 3.3 Plaque characteristics assessed using OFDI

The results of the qualitative and semiquantitative analysis of various coronary plaque characteristics using OFDI are compared in Table 3. The mean lipid index (818 ± 806 vs 1574 ± 891) and macrophage grade (13.5 ± 5.9 vs 19.3 ± 7.4) were significantly lower in the EPA group than in the no‐EPA group (*P* = 0.010 and *P* = 0.019, respectively). However, the mean minimum FCT (109.2 ± 55.7 μm vs 81.6 ± 36.4 μm) was higher in the EPA group than in the no‐EPA group (*P* = 0.040).

**Table 3:** Characteristics assessed using optical frequency domain imaging

	No‐EPA (n = 48)	EPA (n = 12)	*P* value
Plaque rupture	5 (10)	1 (8)	0.830
Plaque erosion	4 (8)	1 (8)	1.000
Luminal thrombus	9 (19)	2 (17)	0.868
Lipid‐rich plaque	37 (77)	6 (50)	0.081
Maximum lipid arc (degree)	236 ± 84	161 ± 106	0.011
Lipid length (mm)	6.2 ± 2.6	3.8 ± 2.8	0.007
Lipid index	1574 ± 891	818 ± 806	0.010
Thin‐cap fibroatheroma	20 (42)	3 (25)	0.334
Minimum fibrous cap thickness (μm)	81.6 ± 36.4	109.2 ± 55.7	0.040
Macrophage infiltration	48 (100)	12 (100)	1.000
Macrophage grade	19.3 ± 7.4	13.5 ± 5.9	0.019
Cholesterol crystals	17 (35)	3 (25)	0.734
Microchannels	45 (94)	11 (92)	0.796
Number of microchannels (/section)	0.37 ± 0.30	0.23 ± 0.26	0.063
Calcification	37 (77)	6 (50)	0.081
Maximum thickness of calcification	637 ± 364	743 ± 374	0.510

### 3.4 Multiple logistic regression analyses for lipid index, TCFA, and macrophage invasion

Multiple logistic regression analyses were performed to assess the risk factors for lipid index, TCFA, and macrophage invasion (n = 60). Because LDL cholesterol to HDL cholesterol ratio was negatively correlated with HDL cholesterol concentration (*r* = −0.675, *P* < 0.001), it was excluded from the multiple variable analysis. Triglyceride concentration and prior EPA use were not significantly correlated with HDL cholesterol concentration (*r* = −0.205, *P* = 0.117, and *r* = −0.033, *P* = 0.803, respectively). Prior EPA use and HDL cholesterol concentration were independently associated with lipid index (Table 4), whereas HDL cholesterol concentration was associated with TCFA (Table 5). Moreover, CKD and prior EPA use were associated with macrophage infiltration (Table 6).

**Table 4:** Logistic regression analysis of lipid index

	Analysis				
	Single		Multiple	
	OR (95% CI)	*P* value	OR (95% CI)	*P* value
Age (years)	1.52 (0.50‐4.61)	0.460		
Male	1.33 (0.37‐4.77)	0.658		
Body mass index	6.33 (0.75‐53.5)	0.090		
Diabetes mellitus	1.11 (0.37‐3.31)	0.853		
Hypertension	0.83 (0.19‐3.63)	0.807		
Dyslipidemia	1.37 (0.21‐8.94)	0.742		
Chronic kidney disease (no hemodialysis)	7.21 (0.86‐60.5)	0.069		
Hemodialysis	3.35 (0.38‐30.0)	0.279		
Sleep apnea syndrome	0.46 (0.08‐2.52)	0.370		
Current smoker	0.15 (0.01‐1.50)	0.105		
Family history of coronary artery disease	1.54 (0.15‐15.8)	0.716		
History of PCI or CABG	1.00 (0.33‐2.99)	1.000		
History of myocardial infarction	1.40 (0.44‐4.43)	0.567		
History of TIA or cerebral infarction	0.57 (0.14‐2.42)	0.447		
History of peripheral artery disease	1.54 (0.15–15.8)	0.716		
Prior statin use	1.29 (0.39‐4.25)	0.680		
Prior aspirin use	0.89 (0.28‐2.85)	0.844		
Prior clopidogrel use	1.11 (0.38‐3.24)	0.855		
Prior ACEI or ARB use	0.67 (0.23‐1.97)	0.462		
Prior calcium channel blocker use	1.35 (0.46‐3.97)	0.584		
Prior beta blocker use	0.57 (0.18‐1.77)	0.329		
Prior eicosapentaenoic acid use	0.27 (0.07‐0.99)	0.048	0.19 (0.04‐0.95)	0.043
Prior ezetimibe use	3.35 (0.38–30.0)	0.279		
Hemoglobin (g/dL)	0.44 (0.15‐1.34)	0.148		
HbA1c (%)	2.25 (0.43‐11.8)	0.336		
Glucose (mg/dL)	2.15 (0.60‐7.70)	0.238		
LDL‐C (mg/dL)	2.79 (0.91‐8.50)	0.072		
Triglyceride (mg/dL)	5.67 (1.65‐19.5)	0.006	3.71 (0.87‐15.8)	0.077
HDL‐C (mg/dL)	0.16 (0.05‐0.51)	0.002	0.19 (0.05‐0.73)	0.016
LDL‐C to HDL‐C ratio	4.50 (1.43‐14.2)	0.010		
Ca (mg/dL)	4.67 (1.28‐17.0)	0.020	4.04 (0.83‐19.7)	0.084
Acute coronary syndrome	1.20 (0.28‐5.25)	0.807		
ST‐segment elevation myocardial infarction	1.00 (0.09‐11.7)	1.000		
Non‐ST‐segment elevation myocardial infarction	0.63 (0.13‐3.13)	0.572		

Abbreviations: ACEI, angiotensin‐converting enzyme inhibitor; ARB, angiotensin II receptor blocker; CABG, coronary artery bypass grafting; CI, confidence interval; HbA1c, hemoglobin A1c; HDL‐C, high‐density lipoprotein cholesterol; LDL‐C, low‐density lipoprotein cholesterol; OR, odds ratio; PCI, percutaneous coronary intervention; TIA, transient ischemic attack.

**Table 5:** Logistic regression analysis of thin‐cap fibroatheroma

	Analysis				
	Single		Multiple	
	OR (95% CI)	*P* value	OR (95% CI)	*P* value
Age (years)	2.26 (0.65‐7.86)	0.200		
Male	2.47 (0.60‐10.1)	0.210		
Body mass index	0.34 (0.09‐1.39)	0.133		
Diabetes mellitus	1.51 (0.53‐4.32)	0.446		
Hypertension	0.56 (0.14‐2.21)	0.410		
Dyslipidemia	0.93 (0.14‐6.01)	0.936		
Chronic kidney disease (no hemodialysis)	4.40 (1.15‐16.9)	0.031	4.10 (0.90‐18.7)	0.068
Hemodialysis	2.39 (0.48‐11.8)	0.286		
Sleep apnea syndrome	0.29 (0.03‐2.66)	0.274		
Current smoker	0.52 (0.05‐5.27)	0.576		
Family history of coronary artery disease	1.67 (0.22‐12.7)	0.622		
History of PCI or CABG	1.43 (0.49‐4.19)	0.516		
History of myocardial infarction	3.39 (1.12‐10.3)	0.031	3.30 (0.94‐11.6)	0.062
History of TIA or cerebral infarction	0.41 (0.08‐2.16)	0.292		
History of peripheral artery disease	5.40 (0.53‐55.4)	0.156		
Prior statin use	0.52 (0.16‐1.65)	0.266		
Prior aspirin use	1.10 (0.36‐3.38)	0.872		
Prior clopidogrel use	0.78 (0.28‐2.21)	0.639		
Prior ACEI or ARB use	1.34 (0.47‐3.84)	0.580		
Prior calcium channel blocker use	0.65 (0.23‐1.86)	0.427		
Prior beta blocker use	0.46 (0.14‐1.50)	0.197		
Prior eicosapentaenoic acid use	0.47 (0.11‐1.94)	0.295		
Prior ezetimibe use	2.39 (0.48–11.8)	0.286		
Hemoglobin (g/dL)	3.20 (0.79‐12.9)	0.102		
HbA1c (%)	1.69 (0.59‐4.89)	0.331		
Glucose (mg/dL)	2.58 (0.88‐7.59)	0.086		
LDL‐C (mg/dL)	1.83 (0.64‐5.27)	0.263		
Triglyceride (mg/dL)	2.41 (0.78‐7.48)	0.128		
HDL‐C (mg/dL)	0.14 (0.04‐0.56)	0.005	0.19 (0.05‐0.81)	0.024
LDL‐C to HDL‐C ratio	2.79 (0.90‐8.69)	0.077		
Ca (mg/dL)	2.82 (0.69‐11.5)	0.148		
Acute coronary syndrome	0.64 (0.15‐2.79)	0.555		
ST‐segment elevation myocardial infarction	3.43 (0.29‐40.1)	0.326		
Non‐ST‐segment elevation myocardial infarction	0.24 (0.03‐2.09)	0.194		

Abbreviations: ACEI, angiotensin‐converting enzyme inhibitor; ARB, angiotensin II receptor blocker; CABG, coronary artery bypass grafting; CI, confidence interval; HbA1c, hemoglobin A1c; HDL‐C, high‐density lipoprotein cholesterol; LDL‐C, low‐density lipoprotein cholesterol; OR, odds ratio; PCI, percutaneous coronary intervention; TIA, transient ischemic attack.

**Table 6:** Logistic regression analysis of macrophage accumulation

	Analysis				
	Single		Multiple	
	OR (95% CI)	*P* value	OR (95% CI)	*P* value
Age (years)	3.78 (0.70‐20.5)	0.123		
Male	1.67 (0.48‐5.86)	0.424		
Body mass index	3.56 (0.84‐15.0)	0.085		
Diabetes mellitus	1.29 (0.46‐3.60)	0.631		
Hypertension	0.92 (0.24‐3.60)	0.908		
Dyslipidemia	1.45 (0.22‐9.34)	0.698		
Chronic kidney disease (no hemodialysis)	7.63 (1.50‐38.7)	0.014	9.71 (1.57‐60.1)	0.014
Hemodialysis	1.49 (0.30‐7.33)	0.621		
Sleep apnea syndrome	1.08 (0.20‐5.82)	0.931		
Current smoker	0.33 (0.03‐3.40)	0.354		
Family history of coronary artery disease	1.07 (0.14‐8.17)	0.945		
History of PCI or CABG	0.90 (0.32‐2.52)	0.833		
History of myocardial infarction	2.34 (0.79‐6.93)	0.126		
History of TIA or cerebral infarction	0.25 (0.05‐1.34)	0.107		
Prior statin use	0.91 (0.29‐2.87)	0.876		
Prior aspirin use	0.57 (0.19‐1.71)	0.315		
Prior clopidogrel use	1.00 (0.37‐2.77)	0.993		
Prior ACEI or ARB use	1.13 (0.41‐3.13)	0.821		
Prior calcium channel blocker use	1.14 (0.42‐3.15)	0.796		
Prior beta blocker use	1.29 (0.43‐3.83)	0.650		
Prior eicosapentaenoic acid use	0.16 (0.03‐0.79)	0.025	0.12 (0.02‐0.76)	0.024
Prior ezetimibe use	1.49 (0.30–7.33)	0.621		
Hemoglobin (g/dL)	1.57 (0.48‐5.14)	0.458		
HbA1c (%)	1.96 (0.69‐5.59)	0.208		
Glucose (mg/dL)	2.24 (0.79‐6.32)	0.128		
LDL‐C (mg/dL)	2.19 (0.68‐7.10)	0.190		
Triglyceride (mg/dL)	2.63 (0.92‐7.49)	0.070		
HDL‐C (mg/dL)	2.95 (0.98‐8.89)	0.055		
LDL‐C to HDL‐C ratio	2.94 (0.92‐9.36)	0.068		
Ca (mg/dL)	2.98 (0.81‐10.9)	0.099		
Acute coronary syndrome	1.08 (0.28‐4.21)	0.908		
ST‐segment elevation myocardial infarction	2.22 (0.19‐25.9)	0.524		
Non‐ST‐segment elevation myocardial infarction	0.78 (0.16‐3.82)	0.758		

Abbreviations: ACEI, angiotensin‐converting enzyme inhibitor; ARB, angiotensin II receptor blocker; CABG, coronary artery bypass grafting; CI, confidence interval; HbA1c, hemoglobin A1c; HDL‐C, high‐density lipoprotein cholesterol; LDL‐C, low‐density lipoprotein cholesterol; OR, odds ratio; PCI, percutaneous coronary intervention; TIA, transient ischemic attack.

### 3.5 Correlations of lipid index, TCFA, and microchannels with macrophage grade

Significant correlations were observed between lipid index, FCT, and macrophage grade. Lipid index was positively correlated with macrophage grade (*r* = 0.803, *P* < 0.001; Figure S2). In contrast, minimum FCT was negatively correlated with macrophage grade (*r* = −0.650, *P* < 0.001; Figure S3). The correlation between macrophage grade and the number of microchannels was not statistically significant (*r* = 0.096, *P* = 0.467). However, in 31 patients in whom the maximum lipid arc was <270°, a weak but positive correlation between macrophage grade and the number of microchannels was observed (*r* = 0.421, *P* = 0.018; Figure S4).

### 3.6 Observer variabilities

Images obtained using OFDI were analyzed by two independent observers. The inter‐observer reliabilities and intra‐observer reproducibilities measured by Pearson's coefficient were *r* = 0.90 and 0.93 for lipid index, *r* = 0.90 and 0.94 for minimum FCT, *r* = 0.89 and 0.92 for macrophage grade, and *r* = 0.92 and 0.95 for the number of microchannels, respectively.

## 4 DISCUSSION

In the present study, the following main observations were of note: First, patients who received EPA therapy had lower lipid burden, higher FCT, and less macrophage accumulation than those who did not receive EPA therapy. Second, in patients undergoing PCI, prior EPA use and HDL cholesterol concentration were independent predictors of lipid index, HDL cholesterol concentration was an independent predictor of TCFA, whereas CKD and prior EPA use were independent predictors of macrophage grade. To the best of our knowledge, this study is the first in‐depth comparison of coronary artery plaques in patients who received and did not receive EPA therapy using propensity score matching and the first analysis of correlations among the characteristics of unstable plaques in patients who underwent PCI using OFDI. These observations further our understanding of the pharmacological effect of EPA therapy, which may have important implications with respect to the management of patients presenting with CAD.

This study suggests that EPA therapy itself is effective for coronary plaque stabilization. As shown in Table 3, we observed that patients who received EPA therapy had lower lipid burden, higher FCT, and less macrophage accumulation than those who did not receive EPA therapy (*P* = 0.010, 0.040, 0.019, respectively). Nonetheless, patient background (including mean LDL cholesterol and triglyceride concentrations), except for EPA/arachidonic acid ratio, was not statistically different between the two groups (*P* = 0.803, Table 1). Watanabe et al. showed that lipid volume and plaque volume reductions with EPA therapy were independent of decreases in LDL cholesterol and triglyceride concentrations,^17^ which is consistent with our results.

### 4.1 Lipid‐rich plaque

An important mechanism of plaque rupture is a large lipid core, which mechanically enhances the tension of fibrous cap covering the lipid core, resulting in plaque disruption.^25^ EPA therapy might reduce lipid core size by inhibiting macrophage accumulation. Wu et al showed that EPA therapy reduced the level of oxidized LDL‐induced cell apoptosis, preventing atherosclerotic progression.^26^ Ferguson et al. reported that EPA attenuated the inflammatory activation of in vitro human adipocytes and reduced lipogenesis.^27^ In the process of atherosclerotic development, lipid‐core enlargement is accelerated by apoptotic macrophage accumulation and elevated chemokine expression followed by intimal recruitment of circulating monocytes.^28^ In the present study, macrophage grade was significantly lower in the EPA group than in the no‐EPA group (Table 3) and was positively correlated with lipid index (Figure S2). Therefore, less macrophage accumulation might contribute to decreased lipid core in the EPA group.

### 4.2 FCT

TCFA is one of the most important characteristics of unstable plaques in the coronary and carotid arteries.^29^, ^30^ Several mechanisms could elucidate the higher FCT in the EPA group than in the no‐EPA group. First, EPA therapy inhibits the ability of macrophages to secrete matrix metalloproteinase (MMP) and monocyte chemotactic protein (MCP)‐1.^31^ As shown in Figure S3, minimum FCT was inversely correlated with macrophage grade. Because the MMPs released by macrophages induce the thinning of fibrous caps of atherosclerotic plaques via collagen breakdown,^32^ less macrophage accumulation in the plaque might contribute to the higher FCT in the EPA group (Table 3). Second, EPA lowered the levels of pentraxin 3, an inflammatory marker associated with TCFA.^33^, ^34^ Yamano et al. showed that EPA administration for 8 months significantly increased the FCT in OCT analysis for non‐culprit plaques with a percent diameter stenosis of 30% to 70%.^35^ Although the mean minimum FCT was higher in the EPA group than in the no‐EPA group (Table 3), EPA therapy was not an independent predictor of TCFA, probably because of the small sample size (Table 5).

### 4.3 Macrophage accumulation

There are several mechanisms for reduced macrophage accumulation by EPA. First, EPA is incorporated into atherosclerotic plaques, and a higher EPA content in the plaques is associated with a reduced number of foam cells and T cells and less inflammation, leading to increased stability.^36^ Second, Yamada et al reported that EPA acts as an anti‐inflammatory agent that reduces the production of pro‐inflammatory eicosanoid mediators from arachidonic acid and decreases adhesion molecule expression.^37^ Furthermore, they showed the in vivo and in vitro inhibition of monocyte adhesion to aortic endothelial cells by EPA. Third, monocyte recruitment into atherosclerotic plaques is also reduced by EPA via favorable alteration of monocyte subsets independent of effects on plasma cholesterol.^38^ Fourth, macrophages extravasating from intraplaque microvessels might be inhibited by EPA via reduction in vascular endothelial growth factor and Flk‐1 receptor expression.^39^, ^40^ In the present study, the mean number of microchannels was lower in the EPA group than in the no‐EPA group, albeit without statistically significant difference (Table 3). These mechanisms might explain the association between EPA therapy and lower macrophage grade.

### 4.4 Other observations

Prior statin use was not significantly associated with plaque stability in the current study (Tables 4, 5, 6). JCAD study showed that if three or more major coronary risk factors are present, the risk for cardiovascular events is 1.3‐fold higher compared to having two or fewer risk factors.^41^ In this study, 35 patients (58%) had three or more major coronary risk factors, which means that the prevalence of high‐risk patients was considerably high. Although such high‐risk patients should have more intensive LDL cholesterol‐lowering therapy,^42^ the mean LDL cholesterol concentration was 94.6 mg/dL in this study. Therefore, prior statin use might not be a statistically significant factor due to inadequate LDL cholesterol‐lowering therapy, or small sample size.

### 4.5 EPA therapy for patients with unstable plaques: clinical perspectives

Several intravascular ultrasound or computed tomography studies have shown that EPA therapy attenuates plaque instability.^15^, ^16^, ^17^, ^18^ In the present study, multiple regression analyses indicated that previous EPA administration was one of the independent predictors of the characteristics of unstable plaques (Tables 4, 5, 6). The results of this study support the hypothesis that EPA therapy is associated with decreased plaque instability. There exists evidence that EPA therapy improves endothelial function^43^ and reduces the risk of cardiovascular events.^2^ The recently published Reduction of Cardiovascular Events With Icosapent Ethyl–Intervention Trial showed a 25% reduction in primary composite endpoints (coronary revascularization or unstable angina) and a 26% reduction in secondary endpoints (cardiovascular death, myocardial infarction, and stroke).^3^, ^44^ Therefore, besides stringent management of other coronary risk factors, EPA therapy might be effective for the secondary prevention of adverse cardiovascular events and the stabilization of unstable plaques in patients with CAD.

### 4.6 Study limitations

The present study has some limitations. First, this retrospective, cross‐sectional study had a small sample size and was conducted at a single medical center within Japan, where people generally have a higher omega‐3 dietary intake in comparison to many other countries. Its results need to be confirmed by a study that enrolls a larger number of patients. Second, there is an inherent discrepancy between characteristics assessed using OFDI and real histopathological findings, as shown in a previous comparison study.^45^ For example, this study could not fully evaluate the presence of intraplaque microchannels, one of the important features of vulnerable plaques, because the near‐infrared light irradiating from the OFDI catheter cannot penetrate through a lipid‐rich plaque and cannot visualize microchannels outside a lipid‐rich plaque. Therefore, further analyses using OFDI with higher resolution and penetration might enable more detailed assessment of intraplaque microstructures in patients undergoing PCI. Third, we do not have additional information on biochemical markers, including pentraxin 3, MMP, MCP‐1, and high‐sensitivity C‐reactive protein. Fourth, the type of drugs, dosing regimen, and duration of therapy were different in EPA group.

## 5 CONCLUSIONS

This OFDI analysis suggests that EPA therapy is associated with decreased plaque instability in patients with CAD undergoing PCI. Patients with CAD who are at high risk for cardiovascular events should receive EPA therapy as intensive medical management for the stabilization of coronary atherosclerotic plaques.

## CONFLICT OF INTEREST

The authors declare no potential conflict of interests.

## Supporting information


**Figure S1.** Semiquantification of macrophage accumulation by optical frequency domain imaging (OFDI). Representative cross‐sectional images of macrophage accumulation obtained using OFDI with the following grades: A, grade 0, no macrophage; B, grade 1, localized accumulation; C, grade 2, clustered accumulation in <1 quadrant; D, grade 3, clustered accumulation in ≥1 quadrant and <3 quadrants; and E, grade 4, clustered accumulation in ≥3 quadrants.

**Figure S2.** Correlation between lipid index and macrophage grade. Lipid index was positively correlated with macrophage grade (*r* = 0.803, *P* < 0.001).

**Figure S3.** Correlation between minimum FCT and macrophage grade
FCT was negatively correlated with macrophage grade (*r* = −0.650, *P* < 0.001).

**Figure S4** Correlation between the number of microchannels and macrophage grade. The number of microchannels was weakly but positively correlated with macrophage grade (*r* = 0.421, *P* = 0.018; n = 31*). *These 31 patients had a maximum lipid arc of <270°.
