# Supplementary figures and images for: Eicosapentaenoic acid therapy is associated with decreased coronary plaque instability assessed using optical frequency domain imaging
Source: Clin Cardiol. 2019 Apr 23;42(6):618–28. doi: 10.1002/clc.23185 (PMC6553360; doi:10.1002/clc.23185)

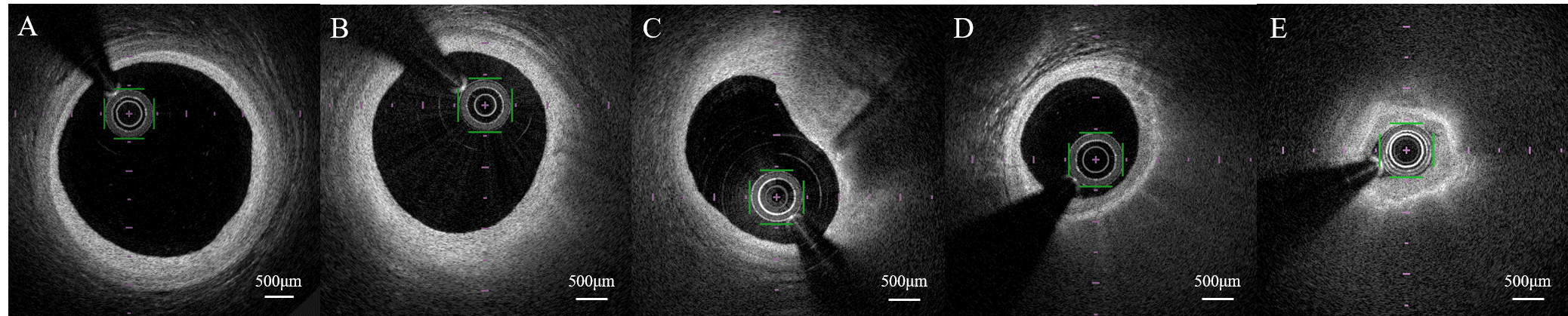

Supplement: Supplementary file 1 — Figure S1. Semiquantification of macrophage accumulation by optical frequency domain imaging (OFDI). Representative cross‐sectional images of macrophage accumulation obtained using OFDI with the following grades: A, grade 0, no macrophage; B, grade 1, localized accumulation; C, grade 2, clustered accumulation in <1 quadrant; D, grade 3, clustered accumulation in ≥1 quadrant and <3 quadrants; and E, grade 4, clustered accumulation in ≥3 quadrants. [file CLC-42-618-s001.tif]

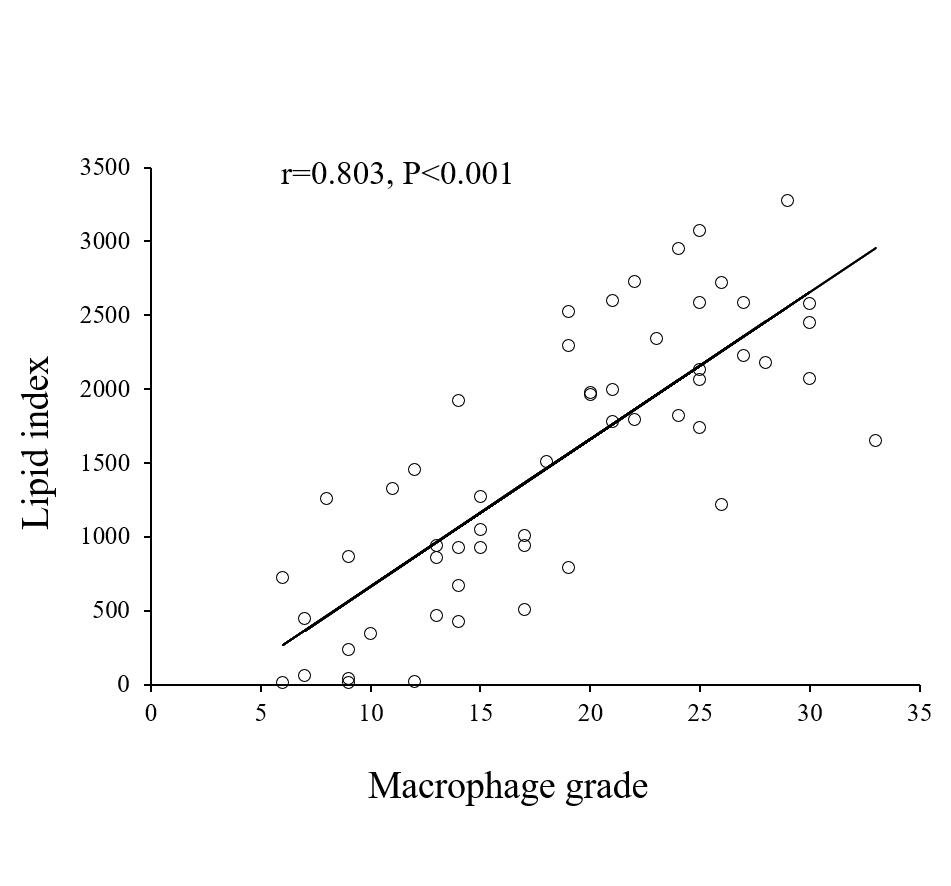

Supplement: Supplementary file 2 — Figure S2. Correlation between lipid index and macrophage grade. Lipid index was positively correlated with macrophage grade (r = 0.803, P < 0.001). [file CLC-42-618-s002.tif]

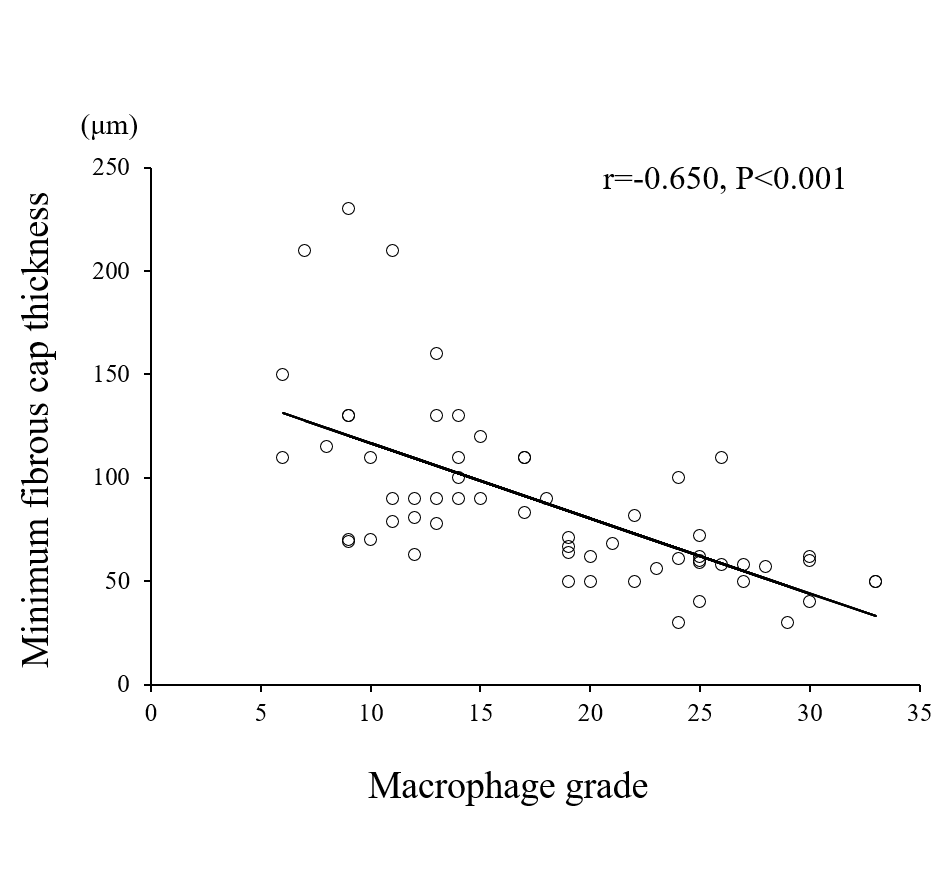

Supplement: Supplementary file 3 — Figure S3. Correlation between minimum FCT and macrophage grade FCT was negatively correlated with macrophage grade (r = −0.650, P < 0.001). [file CLC-42-618-s003.tif]

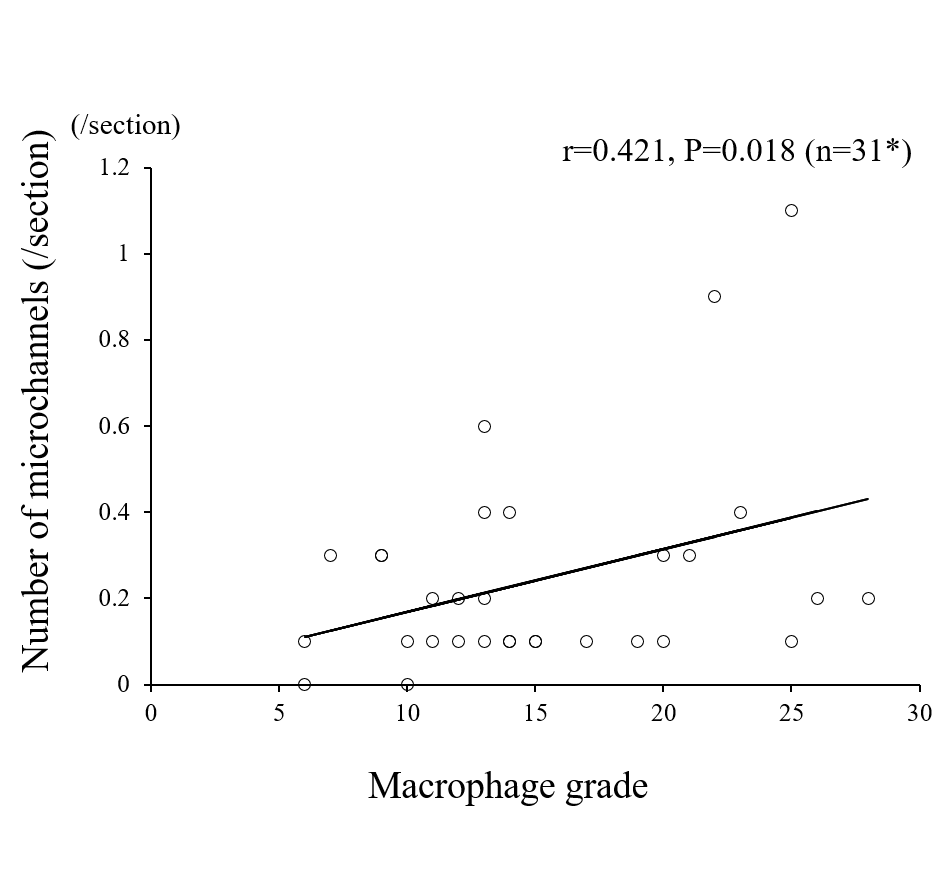

Supplement: Supplementary file 4 — Figure S4 Correlation between the number of microchannels and macrophage grade. The number of microchannels was weakly but positively correlated with macrophage grade (r = 0.421, P = 0.018; n = 31*). *These 31 patients had a maximum lipid arc of <270°. [file CLC-42-618-s004.tif]
